# Exosomal miR-19b-3p communicates tubular epithelial cells and M1 macrophage

**DOI:** 10.1038/s41419-019-2008-0

**Published:** 2019-10-10

**Authors:** Zhi-wei Wang, Xueqiong Zhu

**Affiliations:** 1 Departmant of Obstetrics and Gynecology, The Second Affiliated Hospital of Wenzhou Medical University, Wenzhou, Zhejiang 325027 China; 2 Department of Pathology, Beth Israel Deaconess Medical Center, Harvard Medical School, Boston, MA 02215 USA

**Keywords:** Chronic kidney disease, Acute kidney injury

Dear Editor,

Accumulating evidence has demonstrated that microRNAs (miRNAs), one type of noncoding RNAs, often have short nucleotides (18–25) in length, and negatively regulate gene expression in post-transcriptional level through inhibition of mRNAs or cleavage of mRNAs. Clearly, miRNAs exert their various activities due to the fact that their downstream targets have different biological functions. Recently, miR-19b-3p has been characterized to play a crucial role via different molecular mechanisms in various diseases (Fig. 1). For example, miR-19b-3p is identified to modulate Japanese encephalitis virus (JEV)-mediated inflammation through repression of ring finger protein 11 (RNF11), indicating that miR-19b-3p could be useful for treating viral encephalitis^1^. One study dissected that miR-19b-3p is significantly decreased in exosomes derived from cerebrospinal fluid in the patients with Parkinson’s disease (PD)^2^. However, another study elucidated that plasma miR-19b-3p level is higher in the PD patients compared with healthy persons and multiple-system atrophy (MSA) patients, indicating that miR-19b-3p could be involved in pathophysiology or symptoms of PD and MSA^3^. Notably, miR-19b-3p is reported to be a potential candidate for prediction of autism spectrum disorder (ASD)^4^. Interestingly, miR-19b-3p was reported to play a pivotal role in coordinated guidance of osteoblastic differentiation. Strikingly, miR-19b-3p governs osteogenic differentiation in muscle cells with PDGFRα expression via targeting phosphate and tension homology deleted on chromosome ten (PTEN)^5^. One group revealed that circulating miR-19b-3p could be a potent biomarker for diffuse myocardial fibrosis in hypertrophic cardiomyopathy^6^. In line with this, circulating miR-19b-3p level is correlated to the myocardium level in diabetic cardiomyopathy development, suggesting that this miRNA might be a suitable biomarker in asymptomatic diabetic patients for detection of cardiac dysfunction^7^. Interestingly, miR-19b-3p is involved in liver fibrosis via inhibition of C–C motif chemokine receptor (CCR2). Moreover, miR-19b-3p is dissected to play a potential role in ovarian response to gonadotropins^8^. Low serum expression of miR-19b-3p is observed, and associates with a phenotype of structuring Crohn’s disease^9^. These reports delineate that miR-19b-3p critically participated in the development of human diseases.

**Fig. 1:**
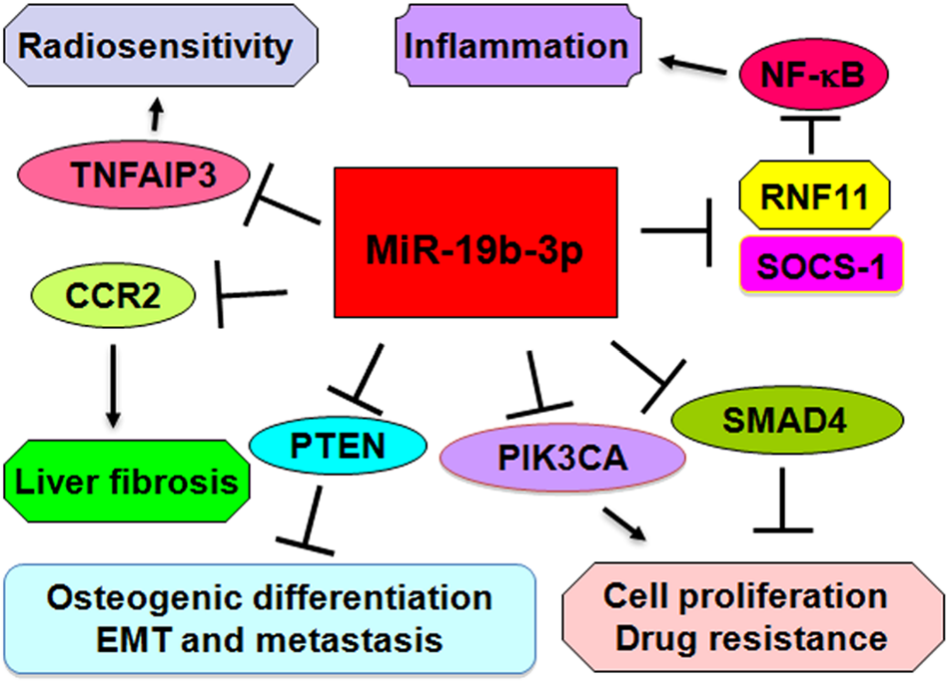
Molecular mechanism of miR-19b-3p is illustrated in cellular functions. MiR-19b-3p has been characterized to play a critical role in multiple human diseases via inhibition of its multiple targets that are involved in cell proliferation, drug resistance, EMT, metastasis, inflammation, and radiosensitivity

Recently, multiple studies have revealed the crucial role of miR-19b-3p in carcinogenesis and progression. Moreover, miR-19b-3p suppresses cell growth and reverses cell resistance to saracatinib by regulation of PIK3CA in breast cancer^10^. Strikingly, miR-19b-3p enhances cell growth and increases chemoresistance to oxaliplatin-based drugs through inhibition of SMAD4 in colon cancer. Intriguingly, cancer stem cell exosomes of clear-cell renal-cell carcinoma transport miR-19b-3p into cancer cells and trigger EMT (epithelial–mesenchymal transition) and metastasis via suppression of PTEN expression^11^. High expression of miR-19b-3p existed in pancreatic cancer tissues as well as exosome samples derived from serum, which is associated with worse overall survival^12^. Similarly, circulating miR-19b-3p is a novel prospective biomarker in gastric cancer for detection and progression. In addition, miR-19b-3p level in plasma is correlated with prostate tumorigenesis. Furthermore, miR-19b-3p participates in cell radiosensitivity via regulation of TNFAIP3 in nasopharyngeal carcinoma^13^.

Kupffer cells (KCs), which are liver-resident macrophages, are activated and then release several mediators, including proinflammatory and profibrotic factors, due to upregulation of CCR2 ligand CCL2, leading to liver inflammation as well as fibrosis. Inactivation of CCL2 reduces toxic liver injury as well as fibrosis via inhibition of the recruitment of macrophages^14^. CCR2 is identified as a direct downstream molecule of miR-19b-3p, indicating that miR-19b-3p is involved in liver fibrogenesis via regulation of inflammation^14^. Although this study reveals the inflammatory role of miR-19b-3p, the exact molecular mechanisms underlying miR-19b-3p-mediated inflammatory response are largely unclear. Recently, Lv et al. determine the molecular mechanisms by which exosomal miR-19b-3p activates M1 macrophage in tubular epithelial cells (TECs) after kidney injury^15^. They observed that miR-19b-3p is highly expressed in TECs in the acute kidney injury (AKI) mice that are induced due to LPS administration and in kidney exosomes by using an adriamycin (ADR)-mediated chronic kidney injury model^15^. Mechanically, this group elegantly identified that TEC exosomes increase activation of M1 macrophage through miR-19b-3p and NF-κB pathways^15^. Notably, miR-19b-3p in exosomes is discovered to activate NF-κB via direct inhibition of the suppressor of cytokine signaling 1 (SOCS-1) in the macrophage. This study further validated that TEC exosomes activate tubulointerstitial inflammation via miR-19b-3p in mice^15^. Upregulation of exosomal miR-19b-3p is observed in the urinary exosomes from biopsy-proven diabetic nephropathy (DN) patients compared with type II diabetes (T2DM) patients, which is associated with tubulointerstitial inflammation severity^15^.

This interesting study provides molecular insight into the roles of miR-19b-3p in connection between the macrophage and TECs in kidney injury. This finding further advocates miR-19b-3p as a promising target for fighting tubulointerstitial inflammation. However, several critical questions still need to be addressed in follow-up studies. For example, to confirm the physiological role of SOCS-1 in the macrophage, it is better to overexpress or deplete the expression of SOCS-1 in cells to validate its function in M1 macrophage activation. Moreover, it will be better to modulate SOCS-1 and then examine whether miR-19b-3p still activates M1 macrophage. Since the authors clarified that 176 miRNAs were differentially expressed in kidney injury, it remains unclear whether other miRNAs play pivotal roles in macrophage activation during kidney injury. Moreover, other good AKI and chronic kidney disease (CKD) models should be considered to validate the insight of miR-19b-3p/SOCS-1/NF-κB axis in kidney injury. Because miR-19b-3p plays a key role in other diseases including cancer, it is important to note that targeting miR-19b-3p for the treatment of tubulointerstitial inflammation needs further investigation. Although miR-19b-3p has multiple targets that are related to cell death, proliferation, and inflammation, most of the previous studies indicated that it might be a “bad guy” due to its role in promoting inflammation, fibrosis, EMT, and cancer progression. Inhibition of miR-19b-3p might be a promising approach for treating kidney disease; however, its effects on cell proliferation and death need careful consideration. Without a doubt, further investigations are warranted to define whether miR-19b-3p could be a prospective target to combat for kidney disease.

## References

[1] Ashraf U (2016). MicroRNA-19b-3p modulates japanese encephalitis virus-mediated inflammation via targeting RNF11. J. Virol..

[2] Gui Y, Liu H, Zhang L, Lv W, Hu X (2015). Altered microRNA profiles in cerebrospinal fluid exosome in Parkinson disease and Alzheimer disease. Oncotarget.

[3] Uwatoko H (2019). Identification of plasma microRNA expression changes in multiple system atrophy and Parkinson’s disease. Mol. Brain.

[4] Mundalil Vasu M (2014). Serum microRNA profiles in children with autism. Mol. Autism.

[5] Zhu Y (2019). MiR-19b-3p regulates osteogenic differentiation of PDGFRalpha(+) muscle cells by specifically targeting PTEN. Cell Biol. Int..

[6] Fang L (2015). Circulating microRNAs as biomarkers for diffuse myocardial fibrosis in patients with hypertrophic cardiomyopathy. J. Transl. Med..

[7] Copier CU, Leon L, Fernandez M, Contador D, Calligaris SD (2017). Circulating miR-19b and miR-181b are potential biomarkers for diabetic cardiomyopathy. Sci. Rep..

[8] Xie S, Batnasan E, Zhang Q, Li Y (2016). MicroRNA expression is altered in granulosa cells of ovarian hyperresponders. Reprod. Sci..

[9] Lewis A (2015). Low serum levels of MicroRNA-19 are associated with a stricturing Crohn’s disease phenotype. Inflamm. Bowel Dis..

[10] Jin J (2018). miR-19b-3p inhibits breast cancer cell proliferation and reverses saracatinib-resistance by regulating PI3K/Akt pathway. Arch. Biochem Biophys..

[11] Wang L (2019). CD103-positive CSC exosome promotes EMT of clear cell renal cell carcinoma: role of remote MiR-19b-3p. Mol. Cancer.

[12] Zou, X. et al. Identification of a six-miRNA panel in serum benefiting pancreatic cancer diagnosis. *Cancer Med.* **8**, 2810–2822 (2019). 10.1002/cam4.2145 PMC6558458 31006985

[13] Huang T (2016). MicroRNA-19b-3p regulates nasopharyngeal carcinoma radiosensitivity by targeting TNFAIP3/NF-kappaB axis. J. Exp. Clin. Cancer Res..

[14] Lan T (2018). Sphingosine kinase 1 promotes liver fibrosis by preventing miR-19b-3p-mediated inhibition of CCR2. Hepatology.

[15] Lv, L. L. et al. Exosomal miRNA-19b-3p of tubular epithelial cells promotes M1 macrophage activation in kidney injury. *Cell Death Differ.* (2019). 10.1038/s41418-019-0349-y. [Epub ahead of print]. 10.1038/s41418-019-0349-y PMC7206053 31097789

